# Pre- and postconditioning the heart with hydrogen sulfide (H_2_S) against ischemia/reperfusion injury in vivo: a systematic review and meta-analysis

**DOI:** 10.1007/s00395-017-0664-8

**Published:** 2017-12-14

**Authors:** Qutuba G. Karwi, Justin S. Bice, Gary F. Baxter

**Affiliations:** 1 School of Pharmacy and Pharmaceutical Sciences, Cardiff University, Redwood Building, King Edward VII Avenue, Cardiff, CF10 3NB UK; 2 Department of Pharmacology, College of Medicine, University of Diyala, Diyala, Iraq

**Keywords:** Preconditioning, Postconditioning, Hydrogen sulfide, Ischemia/reperfusion, Systematic review, Meta-analysis

## Abstract

Conditioning-like infarct limitation by enhanced level of hydrogen sulfide (H_2_S) has been demonstrated in many animal models of myocardial ischemia/reperfusion injury (MIRI) in vivo. We sought to evaluate the effect of H_2_S on myocardial infarction across in vivo pre-clinical studies of MIRI using a comprehensive systematic review followed by meta-analysis. Embase, Pubmed and Web of Science were searched for pre-clinical investigation of the effect of H_2_S on MIRI in vivo. Retained records (6031) were subjected to our pre-defined inclusion criteria then were objectively critiqued. Thirty-two reports were considered eligible to be included in this study and were grouped, based on the time of H_2_S application, into preconditioning and postconditioning groups. Data were pooled using random effect meta-analysis. We also investigated the possible impact of different experimental variables and the risk of bias on the observed effect size. Preconditioning with H_2_S (*n* = 23) caused a significant infarct limitation of − 20.25% (95% CI − 25.02, − 15.47). Similarly, postconditioning with H_2_S (*n* = 40) also limited infarct size by − 21.61% (95% CI − 24.17, − 19.05). This cardioprotection was also robust and consistent following sensitivity analyses where none of the pre-defined experimental variables had a significant effect on the observed infarct limitation. H_2_S shows a significant infarct limitation across in vivo pre-clinical studies of MIRI which include data from 825 animals. This infarct-sparing effect is robust and consistent when H_2_S is applied before ischemia or at reperfusion, independently on animal size or sulfide source. Validating this infarct limitation using large animals from standard medical therapy background and with co-morbidities should be the way forward.

**Electronic supplementary material:**

The online version of this article (10.1007/s00395-017-0664-8) contains supplementary material, which is available to authorized users.

## Introduction

Re-establishing coronary blood flow by either mechanical (primary percutaneous coronary intervention) and/or pharmacological (thrombolytic agents) treatment is essential to limit myocardial damage following acute myocardial ischemia. In industrialised countries, there has been a significant improvement in surgical practice and standard care with an estimate of only one out of four patients with acute ischemic heart attack admitted to early reperfusion intervention dying [41]. Despite this improvement in the survival rate following heart attack, there has also been a considerable increase in long-term co-morbidity and mortality in those patients, which is often a function of the primary infarction. This emphasises the urgent need for treatments which have a therapeutic value for patients with ischemic heart disease. Although an enormous number of mechanical and pharmacological interventions have reported promising infarct-limiting effects experimentally, none has successfully been clinically translated since the first experimental evidence of infarct limitation by ischemic conditioning was reported by Murry et al. [43]. The reasons behind this failure have been discussed in a number of recent reviews and position papers [18, 20, 23] which emphasised three main issues regarding pre-clinical studies. First, there is a “disconnection” between the preclinical and the clinical studies. The complexity of the clinical situation for ischemic heart disease patients needs to be reflected in pre-clinical investigations. This includes common co-morbidities and co-medications which most patients have and are known to modify the response to many cardioprotective manoeuvres experimentally [22, 25]. Second, poor reporting of pre-clinical study methodology and protocols could potentially lead to unnecessary clinical trials [2, 22]. Third, there has been a growing emphasis on interrogating the literature and the careful examination for the pre-clinical evidence using comprehensive, unbiased approaches before conducting any clinical trial [7, 21, 22].

In 1989, hydrogen sulfide (H_2_S) was first detected in rat brain [61], after long being recognised as a toxic gas. It is now recognised as one of the gasotransmitters family along with nitric oxide (NO) and carbon monoxide (CO). There is an increasing body of evidence demonstrating an essential role of H_2_S in health and disease [60]. Experimentally, enhanced levels of H_2_S have been shown to elicit infarct-limiting effect against myocardial ischemia/reperfusion injury (MIRI) in mouse [31], rat [33], rabbit [38] and pig [45]. Promisingly, SG1002, a novel H_2_S prodrug, has recently successfully completed a phase I clinical trial showing a promising margin of safety in failing heart patients [48]. The cardioprotective mechanism(s) by which H_2_S induces its cardioprotection are not fully understood. However, there is general consensus that it is mainly through either activating the reperfusion injury salvage kinase (RISK) pathway, promoting endogenous antioxidant capacity or preserving mitochondrial integrity [5, 14]. Different approaches have been used to enhance H_2_S level in vivo with either conventional inorganic sulfide salts, organic H_2_S donors or phosphodiesterase inhibitors, which we are going to collectively term “H_2_S boosters” in this analysis. These approaches have been shown to limit various markers of MIRI ex vivo and in vivo.

We conducted a comprehensive systematic review and meta-analysis to evaluate the effect of H_2_S on acute myocardial infarction across the in vivo MIRI preclinical studies. In addition, we also performed an additional analysis to provide further insights into the external validity and how the observed infarct limitation by H_2_S could be influenced by different experimental models or pharmacological approaches. Furthermore, we investigated internal validity of our finding and how reporting quality of included studies and publication bias could have an impact on the results and the general conclusion of our study.

## Methodology

### Systematic review and data collection

The systematic review was conducted according to the preferred reporting items for systematic review and meta-analysis (PRISMA) guideline [40]. JSB and QGK performed the literature search of the electronic databases Embase, Medline and Web of Science using selected keywords and MeSH terms where appropriately specific to each database (see the supplementary material).

### Inclusion/exclusion criteria

**Fig. 1:**
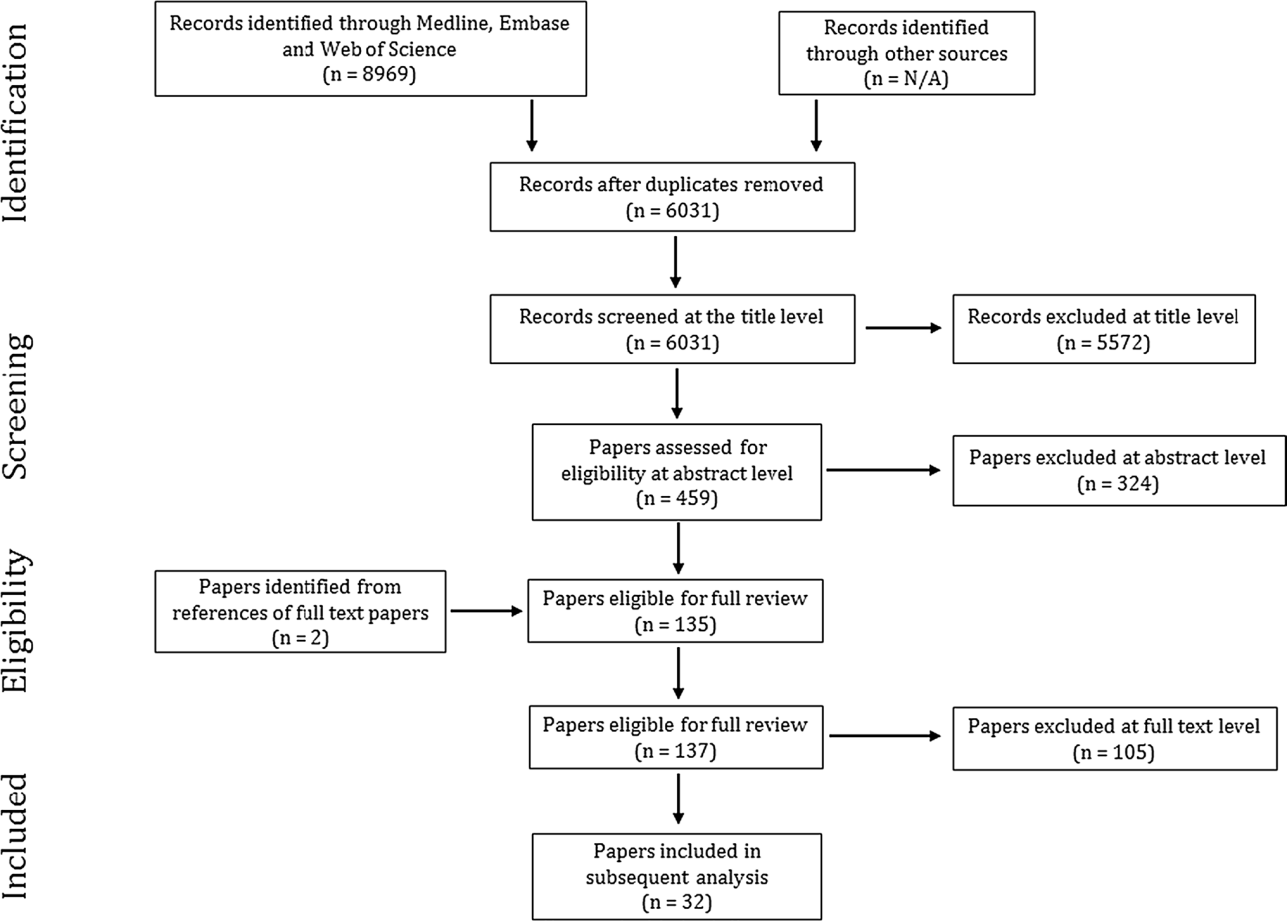
PRISMA diagram of systematic review and data selection at different stages

The search included the literature that investigated the effect of H_2_S on infarct size in in vivo models of MIRI, published between January 01, 2005 and December 16, 2016 considering that the first in vivo report was published in 2006 by Sivarajah et al. [55]. The search only included studies which are available in English. Publications were independently retrieved from the electronic literature and checked for duplication (Fig. 1). The search results were then subjected to the inclusion criteria (Table 1a). Inclusion criteria were developed in accordance with PICOS approach [44]. Reports were included if they characterised the effect of a H_2_S booster (pre- and/or post-ischemia) versus vehicle or no treatment on the infarct size following MIRI in vivo. Studies were excluded at this stage if there was no documented reperfusion phase or if the coronary artery occlusion was permanent. Reports were also excluded where H_2_S treatment was continued throughout ischemia/reperfusion protocol, throughout reperfusion phase, or given more than 10 min after the commencement of reperfusion. In vitro or ex vivo studies were also excluded. Disagreements between the primary reviewers were resolved by secondary reviewer (GFB). Studies employing genetically modified animals or animals with co-morbidity, such as diabetes, heart failure, or high blood pressure, were excluded. Experimental studies where an H_2_S booster was concomitantly administered with other pharmacological treatment, whether it is known for its cardioprotective properties or not, were also not included.

**Table 1:** Lists of (a) inclusion criteria and (b) critical appraisal tool

(a) Inclusion criteria 1. In vivo investigation 2. Documented duration of ischemia and reperfusion 3. Documented time and dose of the exogenous H_2_S booster(s) 4. Infarct size determined by a recognised method
(b) Critical appraisal checklist 1. Characteristics of the animal model (age/weight/sex) 2. Whether the animals were randomly assigned for the control or the treatment group 3. Details about the H_2_S enhancer used including its name, source, dose, route of administration and the time of intervention 4. Whether the experimental protocol is clearly reported including the duration of ischemia and reperfusion and the end point of interest 5. Infarct size determination is clearly detailed 6. Evaluation of the study design including group size and the statistical power 7. Whether a blind-approach of analysis was adopted by the experimentalist at any stage to carry out the measurements and/or to analyse the data 8. Whether the data were statistically analysed using an appropriate test 9. Whether data interpretation was precise and supports the study conclusion 10. Whether study limitations and/or conflicts of interest were clearly documented

### Critical appraisal

The quality and rigour of studies were examined using the critical appraisal tool (Table 1b) which allowed unbiased, comprehensive evaluation of the studies at full-text level. Corresponding authors were contacted by email to enquire about missing information. Thirty-five papers were considered to meet the inclusion criteria and passed critical appraisal to be included in this study (Fig. 1).

### Data extraction and statistical analysis

Our primary outcome was the weighted (unstandardised) mean difference (WMD) in infarct size (IS %) between the experimental group (H_2_S-treated group) and the control group. We identified 65 independent comparisons in the 35 included articles (Table 2). The number of animals in the control group was corrected based on the number of comparisons for each series of experiments (*n*/number of comparisons) [59]. We also identified two experimental variables, namely the animal model size and source of H_2_S, as a secondary outcome which might influence the effect size and heterogeneity.

**Table 2:** Summary of the main characteristics of included pre-clinical studies

	1	2	3	4	5	6	7	8	9	10	11	12	13	14	15	16	17	18	19	20
1	Bibli et al. [3] a	Mouse	M	13–15 weeks	NaHS	Post	(100 µg/kg) as a bolus 10 min before reperfusion	i.v.	30	LAD	2	NR	Ketamine + xylazine + atropine	SEM	6	52.7	4.7	6	20.1	4.3
2	Bibli et al. [3] b	Mouse	M and F	12–15 weeks	NaHS	Post	(100 µg/kg) as a bolus 10 min before reperfusion	i.v.	30	LAD	2	NR	Ketamine + xylazine + atropine	SEM	8	42.2	2.6	8	15.5	1.1
3	Calvert et al. [9] a	Mouse	M	8–10 weeks	Na_2_S	Pre	(100 µg/kg) as a bolus 24 h before ischemia	i.v.	45	LCA	24	R	Ketamine + pentobarbital	SEM	9	48	3	10	26	3
4	Calvert et al. [9] b	Mouse	M	8–10 weeks	Na_2_S	Pre	(100 µg/kg) as a bolus 24 h before ischemia	i.v.	45	LCA	25	R	Ketamine + pentobarbital	SEM	9	25	2	7	18.2	3.3
5	Chatzianastasiou et al. [10] a	Mouse	M	8–12 weeks	Na_2_S	Post	(1 µmol/kg) as a bolus 10 min before reperfusion	i.v.	30	LAD	2	NR	Ketamine + xylazine	SEM	8	37.8	3.3	8	17.8	1.8
6	Chatzianastasiou et al. [10] b	Mouse	M	8–12 weeks	GYY4137	Post	(26.6 µmol/kg) as a bolus 10 min before reperfusion	i.v.	30	LAD	2	NR	Ketamine + xylazine	SEM	8	37.8	3.3	8	19.5	1.4
7	Chatzianastasiou et al. [10] c	Mouse	M	8–12 weeks	Thiovalin	Post	(4 µmol/kg) as a bolus 10 min before reperfusion	i.v.	30	LAD	2	NR	Ketamine + xylazine	SEM	8	37.8	3.3	8	14.4	1.2
8	Chatzianastasiou et al. [10] d	Mouse	M	8–12 weeks	AP39	Post	(250 nmol/kg) as a bolus 10 min before reperfusion	i.v.	30n	LAD	2	NR	Ketamine + xylazine	SEM	8	37.8	3.3	8	16.5	2.3
9	Chen et al. [11] a	Rat	M	250–300 g	hs-MB	Post	6X109/(kg.h) with ultrasonication 5 min before reperfusion until 25 min of reperfusion	i.v.	30	LCA	24	R	Ketamine + pentobarbital	STD	18	41.3	8.6	18	25.3	6.4
10	Chen et al. [11] b	Rat	M	250–300 g	Na_2_S	Post	(100 µg/kg) as a bolus at reperfusion	i.v.	30	LCA	24	R	Ketamine + pentobarbital	STD	18	41.3	8.6	18	26.8	3.9
11	Das et al. [12]	Mouse	M	30–34 g	Ad.PKGIα	Pre	(1.5*109 pfu) 96 h before ischemia	i.v.	30	LCA	24	R	Pentobarbital sodium	SEM	6	37.5	2.2	6	14.1	1.4
12	Donnarumma et al. [13]	Mouse	M	10–14 weeks	Zofenopril	Pre	(10 mg/kg) 8 h before ischemia	PO	45	LCA	24	R	Ketamine + pentobarbital	SEM	12	47.6	4.5	9	33.6	3.7
13	Durrant et al. [15]	Mouse	M	32.2 ± 0.4 g	Na_2_S	Pre	(100 g/kg) before ischemia	i.p.	30	LCA	24	R	Pentobarbital	SEM	4	45	1	4	12.5	0.8
14	Elrod et al. [17] a	Mouse	M	8 weeks	Na_2_S	Post	(50 µg/kg) at reperfusion	i.v.	30	LCA	24	R	Pentobarbital + ketamine	SEM	13	47.9	2.9	8	13.4	1.4
15	Elrod et al. [17] b	Mouse	M	8 weeks	Na_2_S	Post	(50 µg/kg) at reperfusion	i.v.	45	LCA	72	R	Pentobarbital + ketamine	SEM	8	58.3	4.2	8	29.5	4.5
16	Jin et al. [29]	Rat	M	250–300 g	SO_2_ (NaHSO_3_ + Na_2_SO_3_	Pre	(1 µmol/kg) given 10 min before ischemia	i.v.	30	LCA	2	NR	Urethane	SEM	16	43.0	7.0	16	27.5	7.0
17	Kang et al. [31] a	Mouse	M	10–12 weeks	JK-1	Post	(50 µg/kg) at reperfusion	i.v.	45	LCA	24	R	Ketamine + pentobarbital	SEM	12	48	1.8	12	27.5	5.5
18	Kang et al. [31] b	Mouse	M	10–12 weeks	JK-1	Post	(100 µg/kg) at reperfusion	i.v.	45	LCA	24	R	Ketamine + pentobarbital	SEM	12	48	1.8	12	17.2	2.6
19	Kang et al. [31] c	Mouse	M	10–12 weeks	JK-2	Post	(50 µg/kg) at reperfusion	i.v.	45	LCA	24	R	Ketamine + pentobarbital	SEM	12	45.5	3	12	20.5	3.5
20	Kang et al. [31] d	Mouse	M	10–12 weeks	JK-2	Post	(100 µg/kg) at reperfusion	i.v.	45	LCA	24	R	Ketamine + pentobarbital	SEM	12	45.5	3	12	19	3.5
21	Kang et al. [31] e	Mouse	M	10–12 weeks	GYY4137	Post	(50 mg/kg) at reperfusion	i.v.	45	LCA	24	R	Ketamine + pentobarbital	SEM	11	49.5	4.0	10	34.0	4.0
22	Kang et al. [31] f	Mouse	M	10–12 weeks	DDT-2	Post	(1 mg/kg) at reperfusion	i.v.	45	LCA	24	R	Ketamine + pentobarbital	SEM	10	44.0	3.0	10	40.0	4.0
23	Kang et al. [30]	Rat	M	250–300 g	NaHS	Pre	(30 µmol/kg) 30 min before ischemia	i.p.	30	LAD	2	NR	Chloral hydrate	SEM	5	35.0	5.5	5	22.5	6.0
24	Karwi et al. [33] a	Rat	M	300–350 g	GYY4137	Post	(266 µmol/kg) as a bolus 10 min before reperfusion	i.v.	30	LAD	2	NR	Thiobutabarbital	SEM	10	52.5	4.7	8	27.9	3.8
25	Karwi et al. [33] b	Rat	M	300–350 g	GYY4137	Post	(266 µmol/kg) as a bolus 10 min before reperfusion	i.v.	30	LAD	2	NR	Thiobutabarbital	SEM	7	56.8	3.5	7	27.6	2.0
26	Karwi et al. [32] a	Rat	M	300–350 g	AP39	Post	(0.1 µmol/kg) 10 min before reperfusion	i.v.	30	LAD	2	NR	Thiobutabarbital	SEM	10	52.8	3.9	8	43.3	2.5
27	Karwi et al. [32] b	Rat	M	300–350 g	AP39	Post	(1 µmol/kg) 10 min before reperfusion	i.v.	30	LAD	2	NR	Thiobutabarbital	SEM	10	52.8	3.9	8	32.1	3.3
28	Karwi et al. [32] c	Rat	M	300–350 g	AP39	Post	(1 µmol/kg) 10 min before reperfusion	i.v.	30	LAD	2	NR	Thiobutabarbital	SEM	11	53	2.1	8	30.1	2.7
29	Li et al. [36] a	Rat	M	200–250 g	NaHS	Pre	(1.4 µmol/kg) 10 min before ischemia	i.v.	30	LAD	2	NR	Isoflurane	SEM	8	34.8	2.0	8	27.5	2.5
30	Li et al. [36] b	Rat	M	200–250 g	NaHS	Pre	(2.8 µmol/kg) 10 min before ischemia	i.v.	30	LAD	2	NR	Isoflurane	SEM	8	34.8	2.0	8	22.5	0.5
31	Li et al. [36] c	Rat	M	200–250 g	NaHS	Pre	(14 µmol/kg) 10 min before ischemia	i.v.	30	LAD	2	NR	isoflurane	SEM	8	34.8	2.0	8	20.0	2.0
32	Lougiakis et al. [38]	Rabbit	M	2.8–3.1 kg	4-OH-TBZ	Post	(1.79 µmol/kg) as a bolus at 20 min of reperfusion	i.v.	30	LAD	4	NR	Sodium thiopentone	SEM	6	47.9	0.7	6	24.9	0.7
33	Osipov et al. [45]	Pig	M	12 weeks	Na_2_S	Post	(0.2 mg/kg) as a bolus at reperfusion	i.v.	60	LAD	2	NR	Telazol	SEM	6	42.3	5.3	6	16	6.5
34	Predmore et al. [49] a	Mouse	M	8–10 weeks	DATS	Post	(200 µg/kg) as a bolus at reperfusion	i.v.	45	LCA	24	R	Ketamine + pentobarbital	SEM	11	53.8	3	14	24.5	2.5
35	Predmore et al. [49] b	Mouse	M	8–10 weeks	DATS	Post	(200 µg/kg) 22.5 min before reperfusion	i.p.	45	LCA	24	R	Ketamine + pentobarbital	SEM	11	53.8	3	6	18.8	2.0
36	Salloum et al. [51] a	Mouse	M	32.2 ± 0.4 g	Tadalafil	Pre	(1 mg/kg) 1 h before ischemia	i.v.	30	LAD	24	R	Pentobarbital	SEM	6	40.6	2.5	6	13.2	1.7
37	Salloum et al. [51] b	Mouse	M and F	32.2 ± 0.4 g	Tadalafil	Pre	(1 mg/kg) 1 h before ischemia	i.v.	30	LAD	24	R	Pentobarbital	SEM	6	45	2.5	6	18.1	2.1
38	Salloum et al. [52] a	Rabbit	M	2.6–3.2 kg	Cinaciguat	Pre	(1 µg/kg) as a bolus prior to ischemia	i.v.	30	LAD	3	NR	Pentobarbital	SEM	6	37.8	0.7	6	14.1	0.9
39	Salloum et al. [52] b	Rabbit	M	2.6–3.2 kg	Cinaciguat	Post	(10 µg/kg) as a bolus 5 min before reperfusion	i.v.	30	LAD	3	NR	Pentobarbital	SEM	6	37.0	0.5	6	22	2.9
40	Salloum et al. [52] c	Mouse	M	32.2 ± 0.4 g	Cinaciguat	Pre	(10 µg/kg) 30 min before ischemia	i.p.	30	LAD	24	R	Pentobarbital	SEM	6	45.5	5	6	10.2	3.9
41	Salloum et al. [52] d	Mouse	M	32.2 ± 0.4 g	Cinaciguat	Post	(10 µg/kg) as a bolus 5 min before reperfusion	i.v.	30	LAD	24	R	Pentobarbital	SEM	6	43.0	1.5	6	16.5	3.7
42	Salloum et al. [53]	Mouse	M	28–33 g	Beetroot juice	Pre	(10 g/L) in drinking water for 7 days before ischemia	p.o.	30	LAD	24	R	Pentobarbital	SEM	6	46.5	3.5	6	15.8	3.2
43	Sivarajah et al. []55	Rat	M	220–300 g	NaHS	Pre	(3 mg/kg) as a bolus 15 min before ischemia	i.p.	25	LAD	2	NR	Thiopentone	SEM	10	59	3.8	7	44	1.9
44	Sivarajah et al. [54]	Rat	M	250–320 g	NaHS	Pre	(3 mg/kg) as a bolus 15 min before ischemia	i.v.	25	LAD	2	NR	Thiopentone	SEM	12	58.0	3.0	8	45.0	3.0
45	Snijder et al. [56]	Mouse	M	6–8 weeks	H_2_S gas	Pre	(100 ppm) started 30nminutes before ischemia until 5 min of reperfusion	nasal	30	LAD	24	R	Isoflurane	SEM	20	72.5	1.3	21	27.8	0.8
46	Testai et al. [57]	Rat	M	260–350 g	4CPI	Pre	(0.24 mg/kg) 2 h before ischemia	i.p.	30	LAD	2	NR	Pentobarbital	SEM	6	39.0	2.0	6	25.0	3.0
47	Toldo et al. [58]	Mouse	M	32.4 ± 0.9 g	Na_2_S	Pre	(100 µg/kg) 1 h before ischemia	i.p.	30	LCA	24	R	Pentobarbital	SEM	6	44.4	1.6	6	16.3	1.5
48	Xie et al. [63]	Rat	M	270–320 g	ADT	Post	(50 mg/kg) at reperfusion	i.v.	30	LAD	4	NR	Thiobutabarbital	SEM	10	56.4	5.5	10	36.0	3.0
49	Yao et al. [65]	Rat	M	8 weeks	NaHS	Pre	(30 µmol/kg) 10 min prior to ischemia	i.v.	30	LAD	2	NR	Pentobarbital	SEM	20	32.7	12	24	16.5	5.8
50	Yao et al. [66]	Rat	M	250–300 g	NaHS	Pre	(14 µmol/kg/day) for 7 days prior to ischemia	i.p.	30	LCA	2	NR	Chloral hydrate	STD	6	41.6	6.1	6	30.5	4.5
51	Zhao et al. [68] a	Mouse	M	10–12 weeks	8a	Post	(1 mg/kg) as a bolus at 22.5 of ischemia	i.v.	45	LCA	24	R	Ketamine + pentobarbital	SEM	14	46	5.0	14	28.5	6.0
52	Zhao et al. [68] b	Mouse	M	10–12 weeks	8I	Post	(500 µg/kg) as a bolus at 22.5 of ischemia	i.v.	45	LCA	24	R	Ketamine + pentobarbital	SEM	14	51	6.0	14	31.5	4.5
53	Zhao et al. [69] a	Mouse	M	10–12 weeks	NSHD-1	Post	(50 µg/kg) at reperfusion	i.v.	45	LAD	24	R	Pentobarbital + ketamine	SEM	12	51.0	3.0	6	55.0	6.0
54	Zhao et al. [69] b	Mouse	M	10–12 weeks	NSHD-1	Post	(100 µg/kg) at reperfusion	i.v.	45	LAD	24	R	Pentobarbital + ketamine	SEM	12	51.0	3.0	12	32.5	5.0
55	Zhao et al. [69] c	Mouse	M	10–12 weeks	NSHD-2	Post	(50 µg/kg) at reperfusion	i.v.	45	LAD	24	R	Pentobarbital + ketamine	SEM	12	46.5	6.0	12	25.5	4.5
56	Zhao et al. [69] d	Mouse	M	10–12 weeks	NSHD-2	Post	(100 µg/kg) at reperfusion	i.v.	45	LAD	24	R	Pentobarbital + ketamine	SEM	12	46.5	6.0	17	24	5.0
57	Zhu et al. [70]	Rat	M	200–250 g	NaHS	Post	(14 µmol/kg) at reperfusion	i.v.	30	LAD	2	NR	Urethane	STD	12	37.4	3.3	12	19.0	2.0
58	Zhuo et al. [71]	Rat	M	250–300 g	NaHS	Pre	(14 µmol/kg/day) for 6 day prior to ischemia	i.p.	30	LAD	48	R	Chloral hydrate	STD	8	32.7	3.7	8	22.2	5.9

Different letters refer to different studies and experimental groups within each included study (i.e. reference) and have been given according to how these studies or experimental group appear in the included each study following ascending order from A to Z

*M* male, *F* female, *pre* preconditioning, *post* postconditioning, *LAD* left anterior descending, *LCA* left coronary artery, *SEM* standard error of the mean, *STD* standard deviation of the mean

The main characteristics included: (1) study reference; (2) species; (3) gender; (4) age or weight; (5) H_2_S booster; (6) time of intervention; (7); conditioning protocol (pre- or postconditioning dose); (8) route of administration; (9) duration of index ischemia duration (min); (10) coronary artery occluded; (11) reperfusion duration (h); (12) recovery (R) or non-recovery (NR); (13) induction anaesthetic; (14) measure of variance; (15) control group sample size; (16) control group mean infarct size; (17) control group variance; (18) conditioning group sample size; (19) conditioning group mean infarct size; (20) conditioning group variance

Comparisons were divided into two main groups: preconditioning group (pre-H_2_S), where H_2_S booster was given any time before the onset of ischemia, and postconditioning group (post-H_2_S), where H_2_S booster was administrated during regional ischemia or at the commencement of reperfusion. The rationale for grouping the comparisons according to the time of intervention was due to the fact that these two windows of intervention arguably have different clinical applications. For instance, pre-H_2_S could be applied when the onset of ischemia is predictable (planned surgery), while post-H_2_S could be used as adjunctive therapy with reperfusion in STEMI patients.

### Meta-analysis

For each independent comparison, the raw effect size (as a primary outcome) was calculated by subtracting the mean infarct size of the experimental group from the infarct size of the control group along with its correspondent 95% confidence interval (95% CI). We pooled raw effect sizes in each main group using random effect meta-analysis which takes into consideration between-comparison- and within-comparison variations and weights each comparison accordingly. Heterogeneity across different experimental protocols and models, within each main group, was quantified using *I* ^2^ statistics [27, 59]. All analyses were carried out using Review Manager (RevMan 5.3.5 Copenhagen, Denmark: The Nordic Cochrane Centre, The Cochrane Collaboration, 2014).

### Sensitivity analysis

We also carried out subgroup analyses using univariate meta-regression based on pre-defined experimental factors (as a secondary outcome) which might potentially have an impact on the observed effect size of H_2_S and heterogeneity. The percentage of between-comparison variability explained by the variable of interest was evaluated by *I* ^2^ and adjusted *R* ^2^ statistics. The level of significance was adjusted to account for multiple comparisons using the Holm–Bonferroni method [26]. Furthermore, we also tested the robustness of our findings by conducting an additional stratified meta-analysis using standardised mean difference (SMD). SMD represented the mean difference in infarct size between control and H_2_S-treated groups divided by the pooled standard deviation of the mean.

### Risk of bias

We also characterised the quality of study reporting for included studies using a predefined 20-point scoring scale based on the ARRIVE guidelines [34]. This was carried out by JSB and GFB independently and aimed to evaluate the rigour and transparency of included reports. Publication bias, in terms of effect size and degree of precision, was also evaluated independently by QGK and GFB by visual inspection of funnel plot of mean difference (MD) vs standard error of the mean (SD) for all included studies.

## Results

### Study selection process

Our initial search of the databases identified 8969 records (Fig. 1); 2938 duplicates were removed at this stage. 6031 reports were screened independently by QGK and JSB at the title level to check for relevancy to our study scope. 459 reports were considered relevant and screened at the abstract level to investigate if they met the inclusion criteria. As a result, 135 papers passed to the full-text review along with two studies which were identified through “snow-balling” at this stage. These articles were independently critiqued by JSB and QGK using our pre-defined, comprehensive critical appraisal tool. Finally, 32 papers were included in our analysis (Table 2), from which we included 58 controlled comparisons. We then divided the comparisons based on the time of intervention into pre-H_2_S (23 comparisons) and post-H_2_S (35 comparisons) groups.

### Meta-analysis

**Fig. 2:**
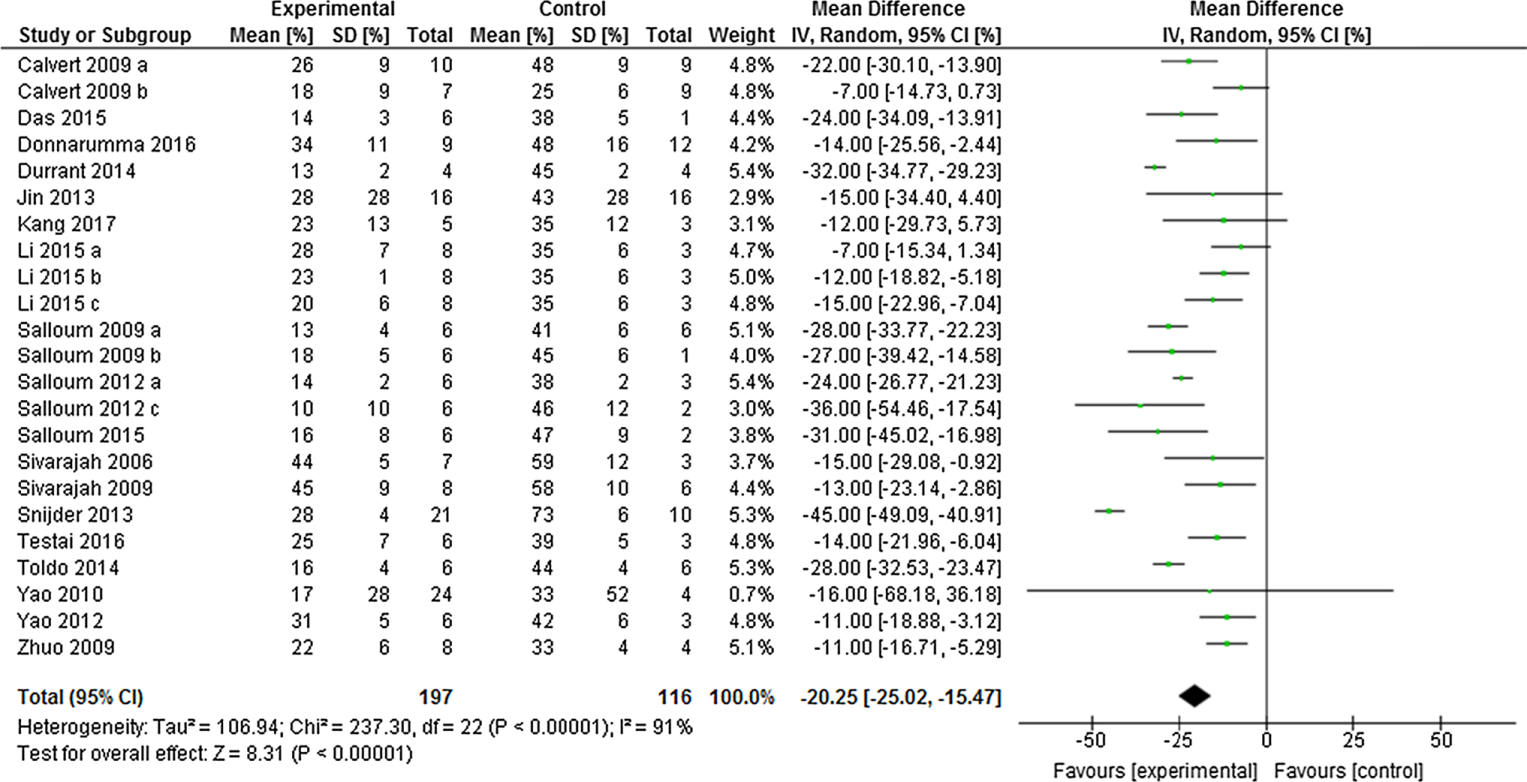
Preconditioning the heart with H_2_S in vivo. Forest plots of meta-analysis of preconditioning the heart with H_2_S boosters on myocardial infarction, pooled using random-effect meta-analysis. Controlled comparisons included data from 116 control animals and 197 treated animals

**Fig. 3:**
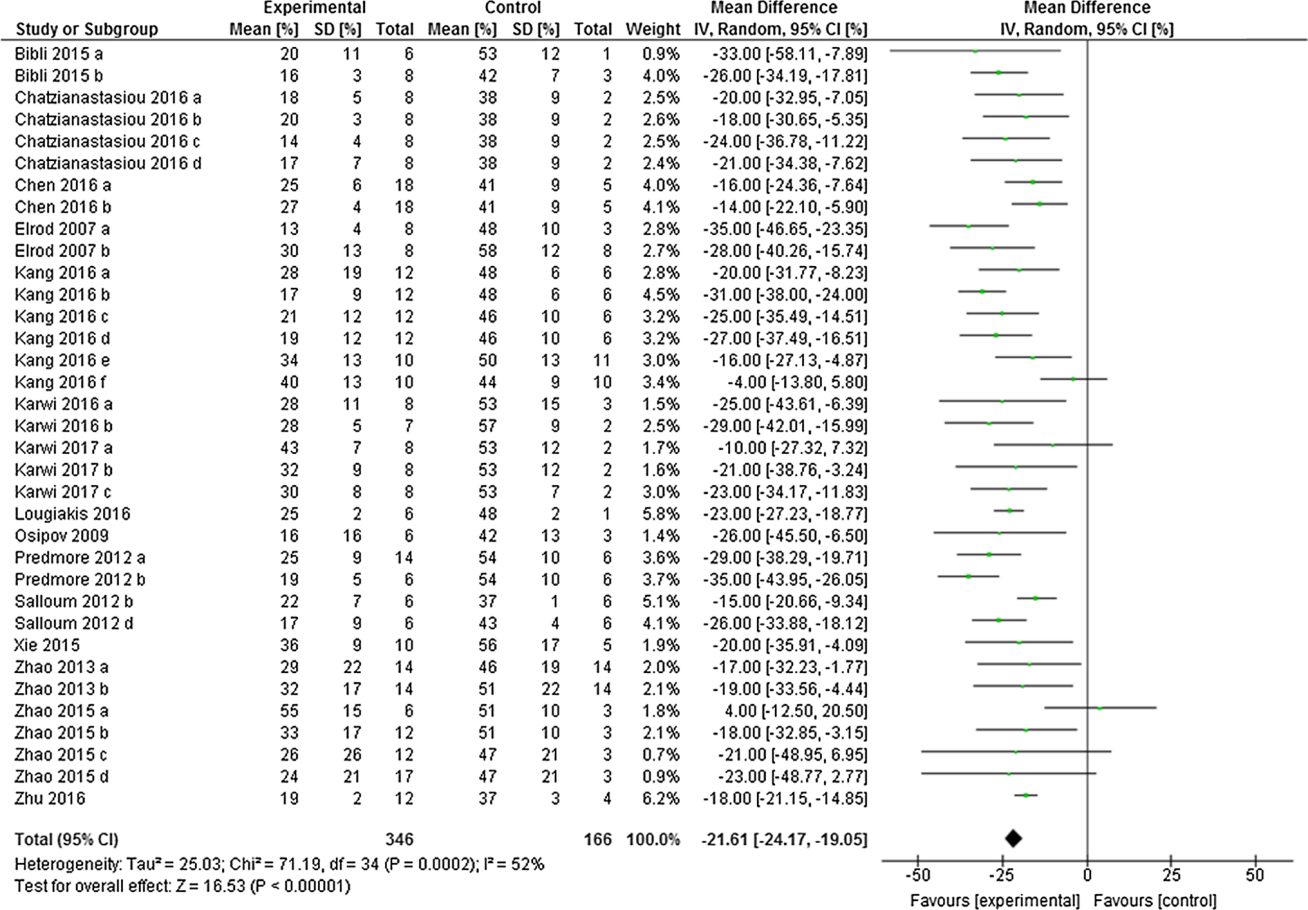
Postconditioning the heart with H_2_S in vivo. Forest plots of meta-analysis of postconditioning the heart with H_2_S boosters on myocardial infarction, pooled using random-effect meta-analysis. Controlled comparisons included data from 346 control animals and 166 treated animals

Preconditioning the heart using H_2_S boosters in vivo caused a significant limitation in infarct size of − 20.25% (95% CI − 25.02, − 15.47; Fig. 2) compared to control (*p* < 0.001, *n* = 23 comparisons). This meta-analysis included data from 116 control animals and 197 animals that received H_2_S boosters before ischemia. This overall effect size was accompanied by a high degree of heterogeneity measured using *I* ^2^ (91%, *p* < 0.001). In the post-H_2_S group, H_2_S also caused a significant infarct limitation of − 21.61% (95% CI − 24.17, − 19.05; Fig. 3), a result which was derived from 166 control animals and 346 H_2_S-treated animals (*p* < 0.001; *n* = 35 comparisons). Likewise, we also observed a high degree of heterogeneity in the post-H_2_S group (*I* ^2^ = 52%, *p* < 0.001).

### Sensitivity analysis

**Fig. 4:**
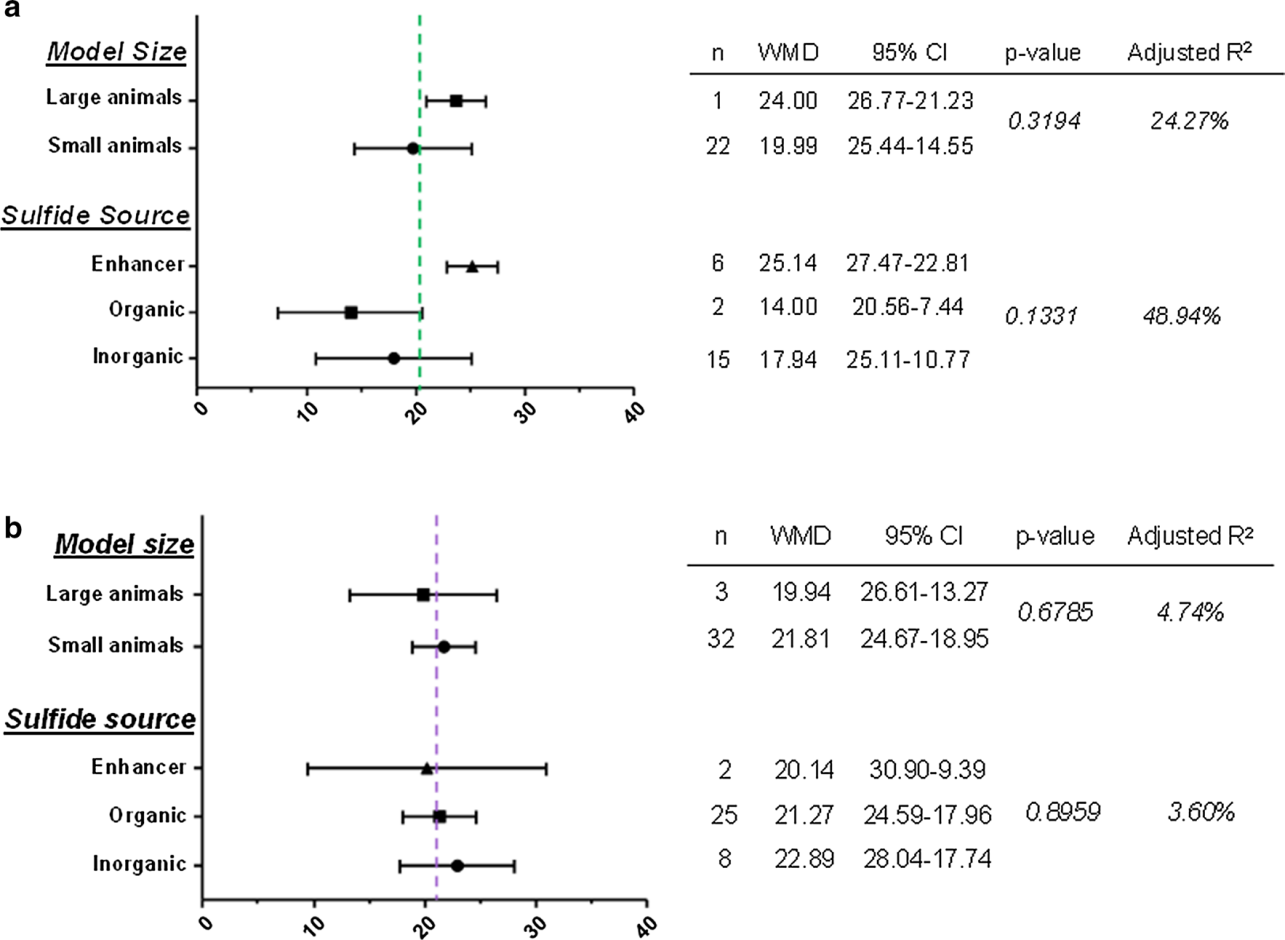
Impact of experimental variables on the overall effect size of **a** preconditioning and **b** postconditioning with H_2_S. Subgroup stratification was used to obtain the weighted mean difference (WMD) along with the corresponding 95% confidence interval (95% CI) followed by meta-regression to obtain the *p* value and avoid false-positive results. Studies that employed mice and rats were grouped as a “small animals” group, while those that used rabbit and pig were grouped as a “large animals” group. Studies were also grouped based on the source of H_2_S to “inorganic” which included sulfide salts and gas, “organic” and “enhancers” which included phosphodiesterase inhibitors. The dotted line indicates the weighted mean difference (WMD). None of the experimental variables had a significant effect on the observed effect size

We also investigated the effect of two crucial experimental variants which might influence the observed effect size, namely the experimental animal size and the source of H_2_S. First, we divided each main group (i.e., pre-H_2_S and post-H_2_S) based on the size of the experimental model into small (mouse and rat) and large models (rabbit and pig). There was no significant difference in the overall effect size between the groups (pre-H_2_S: *p* = 0.3194, adjusted *R* ^2^ = 24.27% (Fig. 4a); post-H_2_S: *p* = 0.6785, adjusted *R* ^2^ = 4.74% (Fig. 4b). We also investigated whether any particular class of H_2_S boosters have an impact on the efficacy of observed infarct limitation. Therefore, we divided each main group based on the class of H_2_S booster into inorganic, organic and enhancer groups. Again, there was no significant difference in the overall effect size between these groups (pre-H_2_S: *p* = 0.1331, adjusted *R* ^2^ = 48.94% (Fig. 4a); post-H_2_S: *p* = 0.8959, adjusted *R* ^2^ = 3.60% (Fig. 4b).

**Fig. 5:**
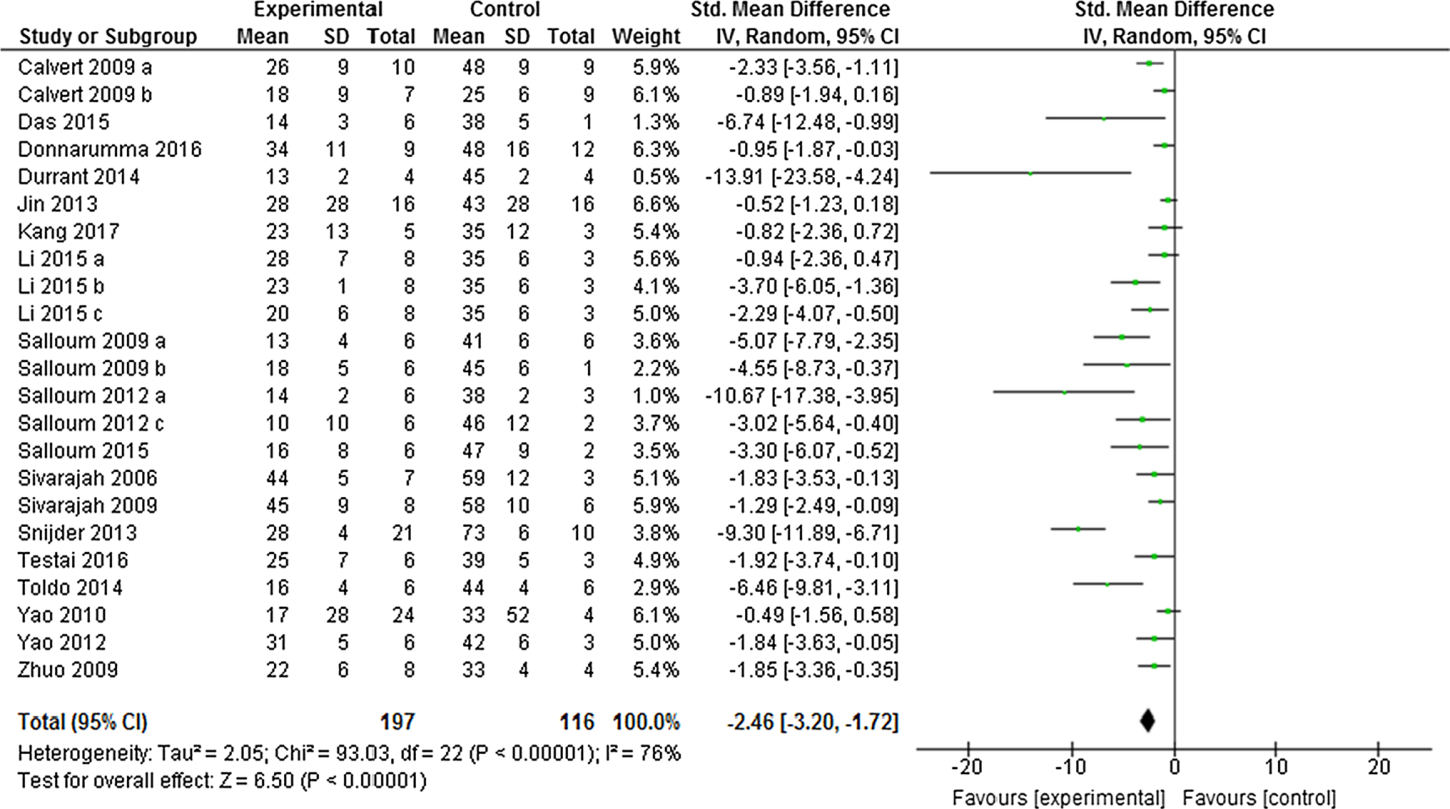
Sensitivity test for the overall infarct limitation by pre-H_2_S in vivo. The overall effect size was calculated using standardised mean difference (SMD), pooled using random-effect meta-analysis. Controlled comparisons included data from 116 control animals and 197 treated animals

**Fig. 6:**
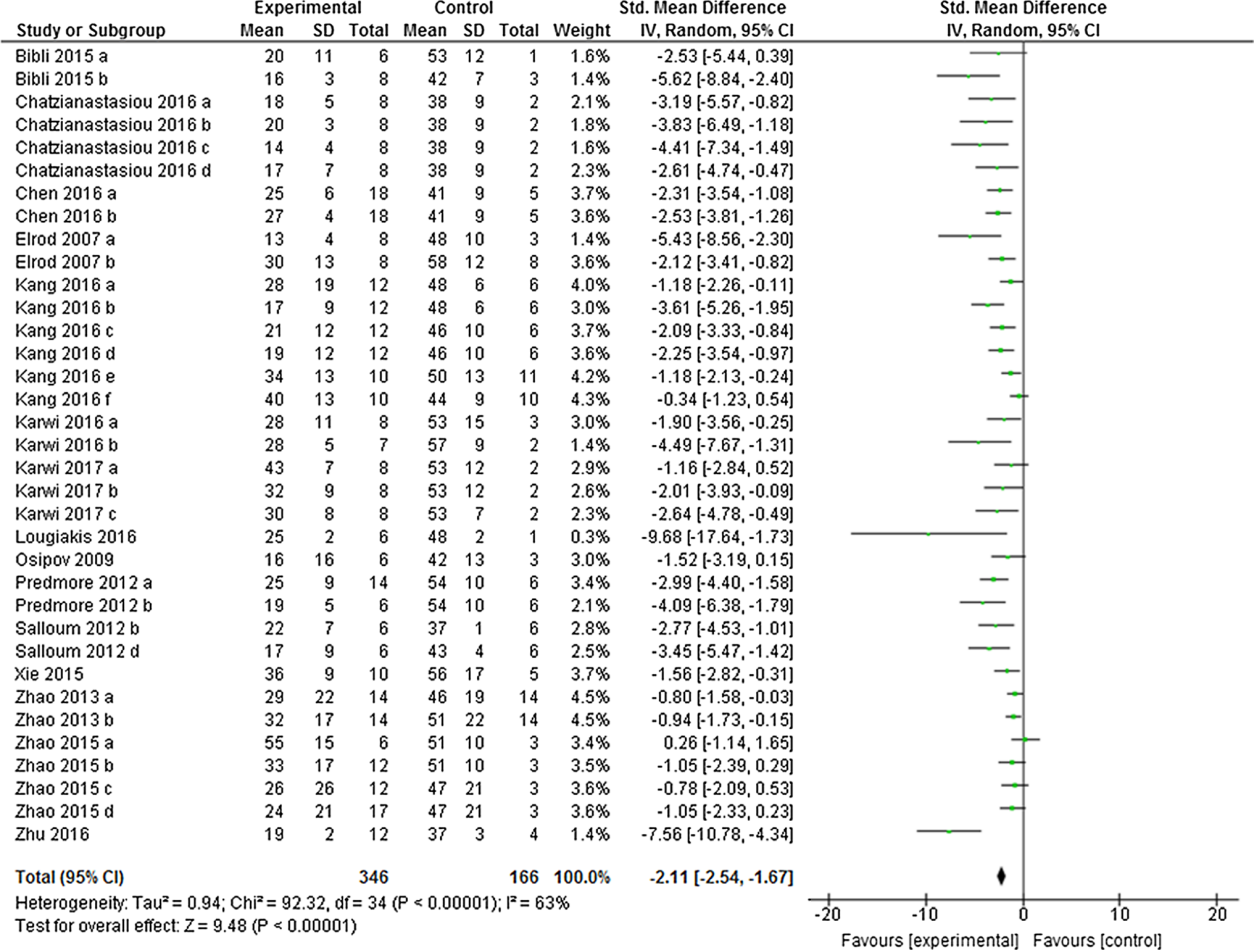
Sensitivity test for the overall infarct limitation by post-H_2_S in vivo. The overall effect size was calculated using standardised mean difference (SMD), pooled using random-effect meta-analysis. Controlled comparisons included data from 346 control animals and 166 treated animals

We examined the robustness of our findings by re-running our meta-analysis using SMD instead of WMD. Interestingly, the results were similar in both cases and H_2_S, again, showed infarct limitation. H_2_S-induced preconditioning limited infarct size by − 2.46% (95% CI − 3.20, − 1.72, *p* < 0.001, Fig. 5) compared to control group with a similar degree of heterogeneity (*I* ^2^ = 76%). Likewise, postconditioning the heart with H_2_S boosters reduced myocardial infarction by − 2.11% (95% CI − 2.54, − 1.67, *p* < 0.001, Fig. 6) compared to the control heart with a similar degree of heterogeneity (*I* ^2^ = 63%).

### Risk of bias

**Fig. 7:**
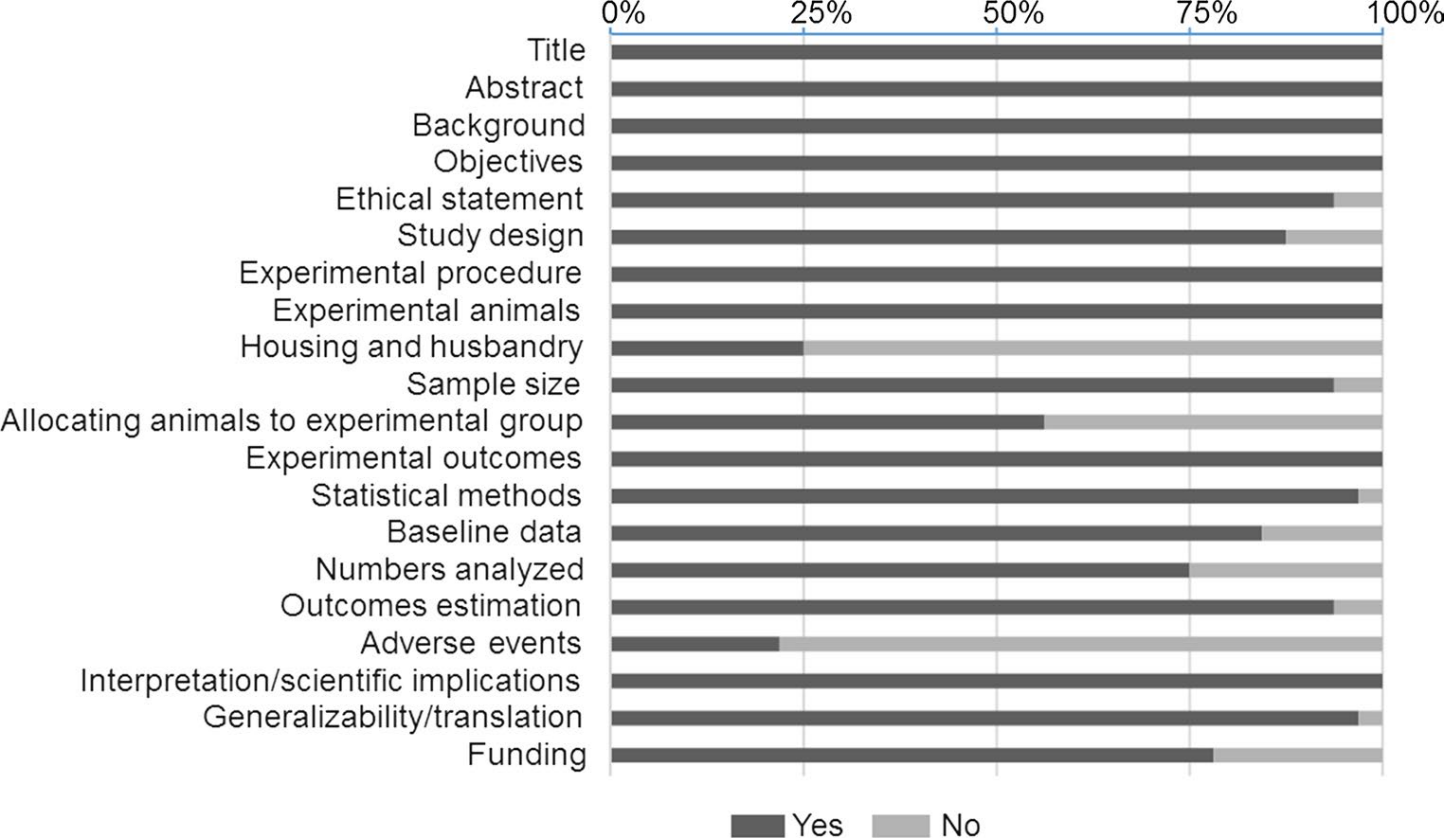
Study reporting quality assessment. The research quality of included studies were evaluated independently by two reviewers according to the quality of study reporting using our pre-defined 20-item quality scoring system. Data were reported as a percentage for each quality item

**Fig. 8:**
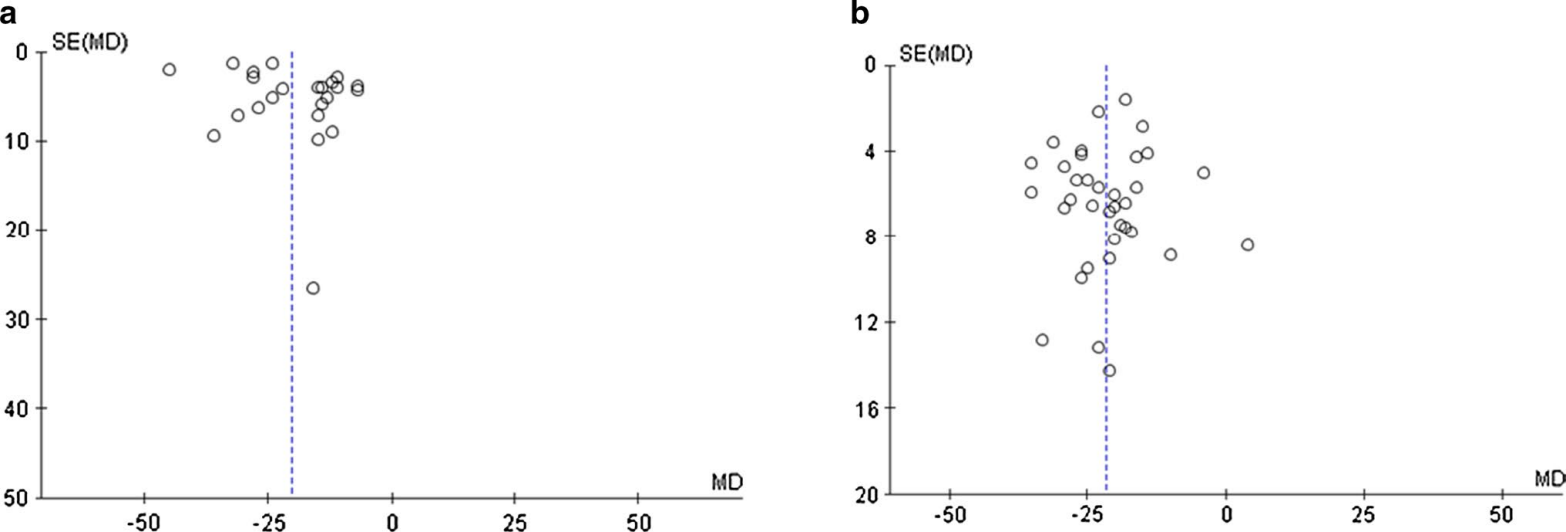
Evaluation of publication bias. A funnel plot showing the precision of the effect estimate in **a** preconditioning group and **b** postconditioning group. The dotted line indicates the weighted mean difference (MD). *SE* standard error

We used a 20-point scoring scale to evaluate the quality of study reporting for included papers derived from ARRIVE guideline (Fig. 7). Included papers scored a median of 17 out of 20 with an interquartile range of 3. We also assessed the publication bias for the included papers by plotting the effect size (WMD) of each controlled comparison against its SD for pre-H_2_S and post-H_2_S groups using funnel plot (Fig. 8). Visual inspection of funnel plots showed that there might be underrepresentation of studies with negative or small effects. Furthermore, we also noticed that there were a few studies with moderate variance among included studies.

## Discussion

The major findings of our systematic review and meta-analysis are that H_2_S has a consistent and robust infarct-limiting effect against MIRI in pre-clinical studies. This robust effect was comparable when H_2_S boosters were given before the onset of ischemia (preconditioning) or at the time of reperfusion (postconditioning) based on in vivo data from almost 900 animals. This cardioprotection also was independent from the animal size or the class of H_2_S booster.

The mechanism of H_2_S-induced conditioning-like phenomena is not fully understood yet, despite several signalling molecules and pathways have been suggested to play a role. However, we here discussed potential conditioning mechanism(s) of H_2_S based on the in vivo evidence included in this study. We took into consideration the causal and temporal consequences of conditioning events and used a structuring scheme previously proposed by Heusch [24]. This scheme is based on the general consensus that conditioning maneuver triggers a “stimulus” which in turn activates a “mediator” to transfer the cardioprotective signal to its “target”. In fact, H_2_S itself has been demonstrated to be a crucial “chemical stimulus” of ischemic pre- [67] and postconditioning [28] to elicit their infarct-limiting effect. Augmented level of H_2_S activates similar signalling molecules and pathways to act as mediators to transmit its cardioprotective signal to its target(s). These signalling pathways mainly involve activating the RISK pathway components in the first minutes of reperfusion [3, 9, 10, 13, 33, 38, 45, 49, 65]. Notably, the activity of some micro-RNAs, namely micro-RNA-21 [58] and mirco-RNA-1 [30], were also reported to serve as mediators of H_2_S-induced cardioprotection. The key target of H_2_S’s protection is the mitochondria, where the majority of salvage signalling pathways converges. Enhanced H_2_S level protects against myocardial infarction via preserving mitochondrial function [17], maintaining membrane integrity [17, 65], limiting mitochondrial ROS generation [32] and inhibiting the opening of mitochondrial permeability transition pore (PTP) [10, 32, 65]. Moreover, mitochondrial K_ATP_ channel is another target of H_2_S protection [54, 55, 57]. However, the question yet to be answered is how H_2_S triggers these signalling pathways to exert its infarct-limiting effect? It is highly unlikely that H_2_S activates the RISK pathway through a ligand/receptor-based mechanism as H_2_S is a gaseous molecule and not a ligand. The most plausible mechanism could be through inducing post-translation modifications (PTMs). Similar to nitrosylation, sulfhydration (or persulfidation) is a PTM induced by H_2_S which could modify the structure and eventually the function of several proteins and channels. Recently, it has been demonstrated that H_2_S activates PI3K/Akt signalling pathways through sulfhydrating phosphatase and tensin homolog (PTEN) abrogating its inhibitory effect [62]. Furthermore, sulfhydration is demonstrated to modify the activity of mitochondrial K_ATP_ channel, another target of H_2_S [54, 55, 57], and ATP synthase (F_1_F_0_ ATP synthase/complex V) [39], the current proposed main component of PTP, which either is known to protect the mitochondria and eventually limit infarct size. Taken together, the role of sulfhydration in conditioning with H_2_S needs further investigation.

There are a number of important aspects which we observed in our review. Despite highly consistent overall effect size, we noticed a high degree of heterogeneity between the included studies. We conducted subgroup analyses to investigate whether some of the experimental variables which we predefined could influence the observed effect size and/or heterogeneity using meta-regression. Others have previously shown, applying the same approach, that experimental model size could have a significant impact on effect size and heterogeneity observed with meta-analysis. For example, Lim et al. [37] reported that cyclosporine-induced infarct limitation in rodent models was absent in a large model (swine) of MIRI in vivo. Noteworthily, this could potentially explain the neutral clinical data of cyclosporine treatment in STEMI patients [25]. However, Bromage et al. [6] recently showed that the infarct limitation by remote ischemic conditioning manoeuvre was consistent across in vivo studies, independently of the model size. Similarly, we previously demonstrated that enhanced level of nitric oxide (NO) in vivo, using different NO treatments, exerted infarct limitation independently of the model size across (22) pre-clinical studies [4]. Our subgroup analyses showed that model size (rodent vs. non-rodent model) did not have a significant effect on either effect size or heterogeneity of H_2_S treatments in both pre-H_2_S and post-H_2_S groups.

We also assessed whether using different H_2_S boosters as a pharmacological approach to enhance H_2_S level could behave differently in terms of infarct limitation and heterogeneity. There have been number of approaches employed to enhance H_2_S level in vivo to investigate its effect on myocardial infarction. Inorganic sulfide salts, namely NaHS and Na_2_S, were the first class of H_2_S boosters initially utilised to investigate the significance of enhancement H_2_S on myocardial infarction. However, they are impure salts that cause a sharp and short-lasting increase in H_2_S level in vivo which make them unreliable H_2_S boosters. Furthermore, off-target or even toxic effects are highly likely with the burst of H_2_S achieved using sulfide salts due to the fact that H_2_S has a narrow therapeutic window. More stable and controllable organic H_2_S donors have been designed to overcome this limitation and have demonstrated infarct-limiting effect in vivo [10, 33, 57, 68]. Utilising triphenylphosphonium scaffold approach to target the mitochondria, we and others have recently reported infarct limitation in vivo using AP39, a mitochondrial-targeting H_2_S donor [10, 32], which have a significant implication considering the central role of mitochondria in MIRI. In a similar context, we have recently reported that the limit of infarct reduction by different NO donors at reperfusion was consistently comparable [4]. Although there was a pattern of increased efficacy of postconditioning with H_2_S enhancers, there was significant difference in the efficacy of any of the H_2_S booster groups in terms of infarct limitation at the two times of intervention. To note, the number of studies that employed large animal models was less than those that used small animals. Furthermore, we also noticed that the cardioprotective dose of some H_2_S boosters could vary between different animal models. For instance, cardioprotective dose of GYY4137, a slow-releasing H_2_S donor, was (26.6 µmol/kg) in the mouse model [10], while it was 10 times more in rat [33]. There is no obvious reason why the cardioprotection dose of these boosters might vary. However, it has been shown that there is a certain degree of dependency of H_2_S on NO signalling to induce its cardioprotection. Arguably, this dependency on NO seems to be high in mouse [3] and partially fading as the animal size increase, such as in rat [33] until it becomes insignificant in large animals, such as rabbit [3]. Whether this hypothesis explains the variation in the cardioprotective dose of some H_2_S boosters requires further investigation.

We were also interested in assessing the impact of other experimental variables which are also important on the external validity of our findings. As our main aim in this review was to characterise the effect of H_2_S on infarct size across the preclinical studies, we, accordingly, excluded all studies which utilised animals with co-morbidities, co-medications and risk factors such as diabetes, heart failure, hypertension or hypercholesterolemia. Therefore, insufficient number of studies in this review rendered these analyses not applicable. Nevertheless, in vivo preclinical studies utilised animals with co-morbidities which were identified in our literature search are summarised in (Table 3). This table is very helpful and has a considerable value for the field of cardioprotection with H_2_S as a starting point for future investigations characterising the impact of co-morbidities on H_2_S protection. Co-morbidities and risk factors associated with cardiovascular disease are important determinants of the efficacy of any cardioprotective therapy and this has recently been discussed in some position papers by others [8, 20, 21, 23]. There is a significant contrast in the biological milieu between the experimental animals and the patients. The majority of the cardioprotective interventions that have been tested in a “reductionist model” employing young and healthy animals, arguably to effectively control the experimental conditions [50]. However, the vast majority of patients recruited in the randomised clinical trials have co-morbidities and/or risk factors including diabetes, aging, hyperlipidemia and hypertension. These co-morbidities and risk factors are shown to modify the efficacy of several cardioprotective interventions [20, 22]. In addition, the potential impact of background medications on the examined efficacy of cardioprotective therapies is often neglected in the pre-clinical studies, despite the fact that most of the recruited patients are on standard medications. Similarly, current standard care could substantially alter the potency of cardioprotective therapies via either blocking the signalling pathway or elevating the threshold which is needed to produce the cardioprotection [22, 47]. Therefore, clinical translation could be considerably enhanced through conducting future preclinical studies on animals with co-morbidities and from a background of standard medications.

**Table 3:** Summary of pre-clinical studies investigated infarct-limiting effect of H_2_S using animals with co-morbidities

	1	2	3	4	5	6	7	8	9	10	11	12	13	14	15	16	17	18	19	20
1	Gao et al. [19]	Streptozocin-induced diabetic rat	M	250–30 g	NaHS	Pre	(14 µmol/kg) daily for 7 days before ischemia	i.p.	30	LAD	2	NR	Chloral hydrate	STD	6	44.0	7.2	6	31.2	4.7
2	Lambert et al. [35] a	Diabetic (db/db) mouse	M	12 weeks	Na_2_S	Post	(0.05 mg/kg) at reperfusion	i.v.	30	LCA	4	NR	Ketamine + pentobarbital	SEM	7	74.2	3.1	8	62.0	3.0
3	Lambert et al. [35] b	Diabetic (db/db) mouse	M	12 weeks	Na_2_S	Post	(0.1 mg/kg) at reperfusion	i.v.	30	LCA	4	NR	Ketamine + pentobarbital	SEM	7	74.2	3.1	7	56.3	3.4
4	Lambert et al. [35] c	Diabetic (db/db) mouse	M	12 weeks	Na_2_S	Post	(0.5 mg/kg) at reperfusion	i.v.	30	LCA	4	NR	Ketamine + pentobarbital	SEM	7	74.2	3.1	5	60.1	5.0
5	Lambert et al. [35] d	Diabetic (db/db) mouse	M	12 weeks	Na_2_S	Post	(1 mg/kg) at reperfusion	i.v.	30	LCA	4	NR	Ketamine + pentobarbital	SEM	7	74.2	3.1	5	68.2	3.2
6	Lambert et al. [35] e	Diabetic (db/db) mouse	M	12 weeks	Na_2_S	Post	(0.1 mg/kg) at reperfusion	i.v.	30	LCA	24	R	Ketamine + pentobarbital	SEM	10	68.4	1.8	10	53.9	2.0
7	Lambert et al. [35] f	Diabetic (db/db) mouse	M	12 weeks	Na_2_S	Post	(0.1 mg/kg) at reperfusion	i.v.	30	LCA	4	N R	Ketamine + pentobarbital	SEM	6	67.4	4.6	6	55.0	2.4
8	Peake et al. [46] a	Diabetic (db/db) mouse	M	12 weeks	Na_2_S	Pre	(0.1 mg/kg) the day before ischemia	i.v.	30	LCA	2	N R	Ketamine + pentobarbital	SEM	8	73.5	1.8	8	60.0	1.5
9	Peake et al. [46] b	Diabetic (db/db) mouse	M	12 weeks	Na_2_S	Pre	(0.1 mg/kg) daily for 7 days before ischemia	i.v.	30	LCA	2	N R	Ketamine + pentobarbital	SEM	8	73.5	1.8	10	46.5	2.4

Different letters refer to different studies and experimental groups within each included study (i.e. reference) and have been given according to how these studies or experimental group appear in the included each study following ascending order from A to Z

*M* male, *F* female, *pre* preconditioning, *post* postconditioning, *LAD* left anterior descending, *LCA* left coronary artery, *SEM* standard error of the mean, *STD* standard deviation of the mean

The main characteristics included: (1) study reference; (2) species; (3) gender; (4) age or weight; (5) H2S booster; (6) time of intervention; (7); conditioning protocol (pre- or postconditioning dose); (8) route of administration; (9) duration of index ischemia duration (min); (10) coronary artery occluded; (11) reperfusion duration (h); (12) recovery (R) or non-recovery (NR); (13) induction anaesthetic; (14) measure of variance; (15) control group sample size; (16) control group mean infarct size; (17) control group variance; (18) conditioning group sample size; (19) conditioning group mean infarct size; (20) conditioning group variance

Another important experimental variable is gender, taking into consideration the cardioprotection of oestrogen which is mainly mediated by triggering the reperfusion injury salvage kinase (RISK) pathway [42], a common signalling pathway with H_2_S [33]. However, only 9% of included studies employed mixed gender. Another dimension to the reductionist model often employed in the pre-clinical studies is the use of a single therapy which is too simplistic and underestimates the clinical complexity. In the view of the current failure in clinical translation, the use of two or more drugs in what is often called “combination therapy” has been suggested as an alternative approach [22]. Especially, some combination treatments have shown promising benefits in vivo [64] and in human [16]. With the current advanced feasibility in designing H_2_S boosters which target different cellular compartments, it is tempting to suggest that combination therapy of different H_2_S boosters could potentially enhance the efficacy of H_2_S-induced cardioprotection. Especially, different H_2_S boosters signal through different protective mechanisms and could potentially have additive infarct-limiting effect to each other which maximise the beneficial effect [1, 32]. Despite this very tempting idea along with very encouraging experimental data, this concept has not been investigated yet and needs to be conducted in well-designed studies. We have listed H_2_S boosters which we think have potential clinical translatability along with proposed mechanism(s) of cardioprotection (Table 4). This table would have a great value for the field of cardioprotection and very helpful to test the concept of combination therapy in future investigations.

**Table 4:** List of H_2_S boosters with potential clinical translatability and proposed mechanism of infarct limitation

	H_2_S booster	Efficacy to limit infarct size (%)	Proposed mechanism(s)	References
1	GYY4137	31–51	Activates PI3K/Akt/eNOS/GSK-3β signalling pathway	[10, 30, 32]
2	Thiovalin	62	Triggers eNOS/NO signalling pathway	[10]
3	AP39	43–56	Signals independently of cytosolic signalling pathways Limits mitochondrial ROS generation Inhibits Ca^2+^-induced PTP opening in a cyclophilin-D-independent manner	[10, 31]
4	hs-MB	39	Unknown	[11]
5	Ad.PKGIα	62	Activates PKG	[12]
6	Zofenopril	29	Activates eNOS and increases plasma NO level Upregulates the expression of antioxidant enzymes (thioredoxin-1, glutathione peroxidase-1 and sodium dismutase-1)	[13]
7	JK-1	43–64	Unknown	[30]
8	JK-2	55	Unknown	[30]
9	4-OH-TBZ	48	Unknown	[36]
10	DATS	65	Activates eNOS/NO signalling pathway	[46]
11	Tadalafil	68	Activates PKG	[48]
12	Cinaciguat	62–77	Increases PKG activity and CSE expression	[49]
13	Beetroot juice	66	Unknown	[50]
14	4CPI	36	Activates mitochondrial K_ATP_ channel	[54]
15	ADT	36	Activates AMPK and autophagic flux	[60]
16	8a	38	Unknown	[65]
17	8I	38	Unknown	[65]
18	NSHD-1	36	Unknown	[66]
19	NSHD-2	45	Unknown	[66]

We also evaluated the internal validity of included studies including the quality of study reporting and publication bias and how these factors could have an impact on the observed results. The lack of full and comprehensive description of the methodological approach and study design could result in an overestimated effect size. By subjecting the included reports to our reporting quality assessment, included studies generally scored highly which is strengthening the validity of our study and it is due to our stringent inclusion criteria. Nevertheless, there was particularly poor reporting in a number of aspects including reporting any adverse effects (28%), a main determinant in any drug development. Reporting of sample size calculation was also poor (43%) which raises some important question regarding whether the study was sufficiently powered before commencing the experiments or allowed to continue until certain number of animals per group was achieved. Insufficient adherence to good quality research indicators could inevitably lead to false-positive results and overestimation of the effect size. As a consequence, this might subsequently lead to further testing of a particular treatment in clinical trial, as a logical consequence, which would be unethical and unnecessary. Furthermore, low standard study reporting makes it difficult to ascertain whether the study was conducted according to high-quality research standards which eventually assuring that the data are valid. Noteworthily, failing to report a good quality research could possibility account for the observed heterogeneity in this meta-analysis. Nevertheless, the effect size by H_2_S was consistent and robust despite the observed high heterogeneity which is reassuring.

We also investigated the publication bias within the included studies using funnel plot. The visual examination and the distribution of the effect size along with the precision of the measurement suggested that there might be an underrepresentation of studies with neutral or negative effect as well as studies with moderate precision in our analysis. However, it needs to be stressed here that studies with neutral or negative data are often not given priority, if at all, to be submitted for publication by the majority of the research groups especially that it is highly likely that they will be rejected at the peer review stage.

## Limitation

This review has included all studies which met our stringent inclusion criteria. However, we acknowledge that we were limited by not including papers which are not published in English language for a time and financial limitations. We also had to exclude studies with missing data or those which failed our critical appraisal to enhance the validity of this meta-analysis. Furthermore, we could not identify any particular variable behind the high degree of heterogeneity using our pre-defined experimental variables. In addition, we also acknowledge that the ARRIVE guideline was launched in June 2010 in the UK, while significant number of studies included in our analysis were either published before this date or conducted outside the UK.

## Conclusion

This systematic review and meta-analysis shows a robust and highly reproducible infarct-limiting effect of H_2_S against MIRI in pre-clinical studies. This robust effect was similar when H_2_S was administrated before the onset of regional ischemia or at reperfusion despite the observed high heterogeneity which is reassuring. The current feasibility of designing stable and controllable H_2_S boosters and selectively targeting specific cellular compartments offer a unique opportunity to use a combination therapy of different H_2_S boosters, which signal through different cardioprotective mechanisms, as an adjunct to standard reperfusion protocol. The focus of future investigations should be on characterising the observed infarct-sparing effect of H_2_S in large animal with co-morbidities, such as diabetes and age, and from background of the current standard polypharmacy in a well-designed preclinical studies.

## Electronic supplementary material

Below is the link to the electronic supplementary material.

Supplementary material 1 (DOCX 26 kb)
