# Supplementary material for: Pre- and postconditioning the heart with hydrogen sulfide (H2S) against ischemia/reperfusion injury in vivo: a systematic review and meta-analysis
Source: Basic Res Cardiol. 2017 Dec 14;113(1):6. doi: 10.1007/s00395-017-0664-8 (PMC5730622; doi:10.1007/s00395-017-0664-8)
Supplement: Supplementary file 1 — Supplementary material 1 (DOCX 26 kb) [file 395_2017_664_MOESM1_ESM.docx]

**“Basic Research in Cardiology”**

**BRIC-D-17-00349R1**

**Pre- and Post-conditioning the heart with hydrogen sulfide (H_2_S) against ischemia/reperfusion injury *in vivo*: a systematic review and meta-analysis**

**Qutuba G Karwi^a^, Justin S Bice^a^, Gary F Baxter^a^**

^a^ School of Pharmacy and Pharmaceutical Sciences, Cardiff University, UK;

**Address for correspondence:**

Dr Qutuba G Karwi

School of Pharmacy and Pharmaceutical Sciences, Cardiff University

Redwood Building, King Edward VII Avenue

Cardiff CF10 3NB

United Kingdom

Telephone: +44 (0)29 2087 6446

Email: [karwiqg@cardiff.ac.uk](mailto:karwiqg@cardiff.ac.uk)

ORCID: orcid.org/0000-0003-2897-6630

**Supplementary**

Appendix 1: Search items used to search electronic databases Pubmed, Embase and Web of Science

| No | Keyword |
| --- | --- |
| 1 | Sulfide* |
| 2 | Hydrogen sulfide |
| 3 | Hydrogen sulphide |
| 4 | H_2_S |
| 5 | Hydrogen sulfide donor* |
| 6 | Sulfide donor* |
| 7 | H_2_S-releasing |
| 8 | H_2_S-generating |
| 9 | H_2_S donating |
| 10 | H_2_S donor* |
| 11 | Polysulfide* |
| 12 | NaHS |
| 13 | Sodium hydrosulfide |
| 14 | Na_2_S |
| 15 | Sodium sulfide |
| 16 | GYY* |
| 17 | [morpholin-4-ium 4 methoxyphenyl(morpholino) phosphinodithioate] |
| 18 | DATS |
| 19 | Diallyl trisulfide |
| 20 | DADS |
| 21 | Diallyl disulphide |
| 22 | 4CPI |
| 23 | 4-carboxyphenyl isothiocyanate |
| 24 | TV |
| 25 | Thiovaline |
| 26 | Garlic |
| 27 | Garlic-derivative* |
| 28 | SG-1002 |
| 29 | IK1001 |
| 30 | AP39 |
| 31 | (10-oxo-10-(4-(3-thioxo-3H-1,2-dithiol-5-yl)- |
| 32 | phenoxy)decyl)triphenylphosphonium bromide |
| 33 | AP123 |
| 34 | ADT-OH |
| 35 | Anethole dithiolethione |
| 36 | ATB-346 |
| 37 | 2-(6-methoxynapthalen-2-yl)-propionic acid 4-thiocarbamoyl phenyl ester |
| 38 | Thioglycine |
| 39 | Allitridum |
| 40 | #1 OR #2 OR #3 OR #4 OR #5 OR #6 OR #7 OR #8 OR #9 OR #10 OR #11 OR #12 OR #13 OR #14 OR #15 OR #16 OR #17 OR #18 OR #19 OR #20 OR 21 OR #22 OR #23 OR #24 OR #25 OR #26 OR #27 OR #28 OR #29 OR #30 OR #31 OR #32 OR #33 OR #34 OR #35 OR #36 OR #37 OR #38 OR #39 |
|  |  |
| 41 | Infarct* |
| 42 | Myocard* |
| 43 | Cardio* |
| 44 | Cardiac |
| 45 | Heart |
| 46 | #41 OR #42 OR #43 OR #44 OR #45 |
|  |  |
| 47 | isch* |
| 48 | Reperfusion |
| 49 | Isch* reperfusion injury |
| 50 | I/R injury |
| 51 | IRI |
| 52 | Reperfusion injury |
| 53 | #47 OR #48 OR #49 OR #50 OR #51 OR #52 |
|  |  |
| 54 | administ* |
| 55 | suppl* |
| 56 | exogenous* |
| 57 | oral* |
| 58 | parentral* |
| 59 | Inhaled |
| 60 | Diet |
| 61 | #54 OR #55 OR #56 OR #57 OR #58 OR #59 OR #60 |
|  |  |
| 62 | rodent* |
| 63 | rat* |
| 64 | Mice |
| 65 | Mouse |
| 66 | Murine |
| 67 | Rabbit |
| 68 | Pig |
| 69 | Swine |
| 70 | Cat |
| 71 | Dog |
| 72 | Canine |
| 73 | Feline |
| 74 | Bovine |
| 75 | model* |
| 76 | animals* |
| 77 | animal stud* |
| 78 | in vivo |
| 79 | #62 OR #63 OR #64 OR #65 OR #66 OR #67 OR #68 OR #69 OR #70 OR #71 OR #72 OR #73 OR #74 OR #75 OR #76 OR #77 OR #78 |
|  |  |
| 80 | #40 AND # 46 AND #53 AND #79 |
| 81 | #40 AND # 46 AND #53 AND #61 AND #79 |
|  |  |
| 82 | #40 AND #53 |
|  |  |
